# Genome-Based Analysis of a Sequence Type 1049 Hypervirulent *Klebsiella pneumoniae* Causing Bacteremic Neck Abscess

**DOI:** 10.3389/fmicb.2020.617651

**Published:** 2021-01-18

**Authors:** Peng Lan, Dongdong Zhao, Jiong Gu, Qiucheng Shi, Rushuang Yan, Yan Jiang, Jiancang Zhou, Yunsong Yu

**Affiliations:** ^1^Department of Infectious Diseases, Sir Run Run Shaw Hospital, Zhejiang University School of Medicine, Hangzhou, China; ^2^Key Laboratory of Microbial Technology and Bioinformatics of Zhejiang Province, Hangzhou, China; ^3^Department of Critical Care Medicine, Sir Run Run Shaw Hospital, Zhejiang University School of Medicine, Hangzhou, China; ^4^Department of Infectious Diseases, The First Hospital of Jiaxing, Zhejiang, China

**Keywords:** hypervirulent, *Klebsiella pneumoniae*, neck abscess, ST1049, comparative genomic analysis

## Abstract

Hypervirulent *Klebsiella pneumoniae* (hvKP) has raised grave concerns in recent years and can cause severe infections with diverse anatomic locations including liver abscess, meningitis, and endophthalmitis. However, there is limited data about neck abscess caused by hvKP. A *K. pneumoniae* strain Kp_whw was isolated from neck abscess. We characterized the genetic background, virulence determinates of the strain by genomic analysis and dertermined the virulence level by serum resistance assay. Kp_whw belonged to sequence type (ST) 1049 K locus (KL) 5. Kp_whw showed hypermucoviscosity phenotype and was resistant to ampicillin but susceptible to the majority of the other antimicrobial agents. A pLVPK-like virulence plasmid and a chromosomal ICEKp5-like mobile genetic element were carried by Kp_whw, resulting in the risk of dissemination of hypervirulence. The strain exhibited relative higher level of core genome allelic diversity than accessory genome profile, in comparison to hvKP of K1/K2 serotype. Kp_whw was finally demonstrated as virulent as the ST23 K1 serotype hvKP strain NTUH-K2044 *in vitro*. In conclusion, this work elaborates the genetic background of a clinical hvKP strain with an uncommon ST, reinforcing our understanding of virulence mechanisms of hvKP.

## Introduction

Hypervirulent *Klebsiella pneumoniae* (hvKP) has raised grave concerns in recent years due to its extraordinary invasiveness and hypervirulence, resulting in considerable risks for morbidity and mortality (Shon et al., 2013). Asia is the epidemic area for hvKP. K1/K2 accounted for 9.8% of all *K. pneumoniae* isolates in stools from healthy Chinese individuals in Asia countries (Lin et al., 2012). In recent years, hvKP has been increasingly reported in other continents. The estimated prevalence of hvKP in Canada and the United States was 8.2 and 6.3%, respectively (Peirano et al., 2013; Chou et al., 2016). According to different areas, the mortality of hvKP infections ranged from 29.2 to 55.1% (Lin Y.T. et al., 2010; Rafat et al., 2018; Namikawa et al., 2019). hvKp infection is often manifested as liver abscess (Liu et al., 1986; Siu et al., 2012; Joob and Wiwanitkit, 2017). However, extrahepatic infections have been described for variable anatomic locations including endoophthalmitis (Liu et al., 1986), osteomyelitis (Prokesch et al., 2016) and meningitis (Melot et al., 2016). Originated in the Asian Pacific Rim in 1980s, hvKP has disseminated worldwide.

A number of virulence factors contribute to the pathogenicity of hvKP including lipopolysaccharide (LPS), capsule, fimbriae, siderophores, and virulence plasmids (Paczosa and Mecsas, 2016; Russo and Marr, 2019). Traditionally, hypermucoviscosity, resulted by over expression of capsular polysaccharides, was considered as a critical characteristic for hypervirulence (Catalan-Najera et al., 2017), as the mucoviscous shield significantly increased the resistance to immunological recognition and killing from the host (Lin et al., 2006; Lin J.C. et al., 2010). Recently, virulence plasmids were proved to have potential to transmit virulence genes among *K. pneumoniae* strains (Yang et al., 2019). Virulence plasmids encode two siderophores, aerobactin, and salmochelin, and RmpA (regulator of the mucoid phenotype). The virulence plasmid pK2044 harbored by K1 serotype strain NTUH-K2044 and pLVPK harbored by K2 serotype strain CG43 were well characterized and were reported associated with invasive syndrome. Besides, virulence genes located in chromosome can also be transmitted via integrative and conjugative element (ICE) (Lam et al., 2018). Virulence plasmids and ICEKps both harbor virulence loci encoding variable virulence factors and are involved in horizontal gene transfer (HGT), conferring the global dissemination of hypervirulence.

Clonal complex 23 (CC23) and some selected serotypes (e.g., K1 and K2) are commonly deemed as hypervirulent clones (Struve et al., 2015). Most of hvKP strains of K1 serotype belong to CC23, while K2 serotype strains are genetically more diverse and belong to multiple distinct multilocus sequencing types (MLSTs) (Struve et al., 2015). More than a half of severe *K. pneumoniae* infections such as bacteremia, liver abscess, and invasive extrahepatic infections are caused by K1/K2 serotype strains (Lin Y.T. et al., 2010). In addition, strains of serotype K5, K20, and K54 are also associated with hypervirulence phenotype (Wyres et al., 2020).

Clinically, hvKP usually causes liver abscess with extrahepatic complications including necrotizing fasciitis, bloodstream infection, meningitis, and endophthalmitis (Siu et al., 2012). Patients infected with hvKP are frequently relative healthy and immunosufficient. However, diabetes is a critical risk factor for hvKP infection, partly due to the suppression of the innate immune system (Alba-Loureiro et al., 2007; Hodgson et al., 2015).

Here, we described a potential hypervirulent *K. pneumoniae* strain belonging to ST1049, an uncommon ST, isolated from neck abscess. *K. pneumoniae* of ST1049 was previously reported to cause liver abscess and meningitis, and resulting in poor clinical outcomes (Ku et al., 2017; Zhang et al., 2019). However, no genetic evidence was showed that this infrequent ST was a hypervirulent clone. Hence, we will characterize the strain based on genetic background and virulence profile and thus to refresh our knowledge about hvKP pathogenicity.

## Materials and Methods

### Isolations

The strain was isolated from a male patient diagnosed with neck abscess in 2018. It was identified to the species level via matrix-assisted laser desorption/ionization mass spectrometry and named as Kp_whw. The antimicrobial susceptibilities of the strain were determined by a VITEK-2 compact system and interpreted according to the M100-S26 guideline established by Clinical and Laboratory Standards Institute (CLSI). Since the clinical characteristics were extracted from the electronic record system and were de-identified, informed consent was waived. In addition, 92 hvKP strains of K1/K2 serotype from our previous study (Lan et al., 2020) plus NTUH-K2044 (Wu et al., 2009) and hvKP1 (Russo and Gill, 2013) were employed for comparative genomic analysis.

### Whole Genome Sequencing

The strain was cultured to the mid-logarithmic phase in LB broth at 37°C. Genomic DNA was extracted using the QIAamp DNA Minikit (QIAGEN, Hilden, Germany) and was further purified using the PowerClean DNA cleanup kit (Mo Bio Laboratories, Carlsbad, United States), following the manufacturer’s recommendations. The genome was sequenced on an Illumina HiSeq X Ten platform (Illumina, San Diego, United States) using a paired-end 2 × 150–base pair protocol by Tianke Company (Hangzhou, China). Derived short reads were *de novo* assembled using CLC Genomics Workbench 9.5.1 software. The sequences assemblies reported in this paper have been deposited in the European Nucleotide Archive database (accession nos. PRJEB38367 and PRJEB34922). The NCBI accession number for NTUH-K2044 and hvKP1 are AP006725 and AOIZ00000000.

### Typing and Detection of Virulence Determinants

The virulence genes and *wzi* (a part of K-locus) alleles were identified using Institut Pasteur^1^. Multilocus sequence typing (MLST) analysis for sequence types of *K. pneumoniae* used Institut Pasteur MLST^2^. For MLST, 7 target genes (*gapA*, *infB*, *mdh*, *pgi*, *phoE*, *rpoB*, and *tonB*) were analyzed. To detect the virulence plasmid, the contigs of strain KP_whw were aligned with well-characterized virulence plasmid pLVPK (accession no. AY378100 in GenBank) using the BLAST Ring Image Generator (BRIG, version 0.95) (Alikhan et al., 2011). Putative ICE was identified by ICEberg^3^ and then compared with previously reported ICEKps by Kleborate^4^.

### Phylogenetic Analysis

Ridom SeqSphere + software (version 5.0, Ridom GmbH, Germany) was used for the core genome multi-locus sequence typing (cgMLST) analysis with the whole genome sequence. Compared with common typing method MLST of 7 housekeeping genes or pulse field gel electrophoresis (PFGE), cgMLST scheme shows higher discriminatory power (Pérez-Losada et al., 2018; Gona et al., 2020). cgMLST schemes consist of a fixed set of conserved genome-wide genes. Alleles are used instead of single nucleotide polymorphisms (SNP) or concatenated sequences to mitigate the effects of recombination and to enable for a global and public nomenclature. For *K. pneumoniae*, 2358 target genes named with allelic nomenclature are employed in a cgMLST scheme, with NTUH-K2044 as the reference genome^5^. Similarly, 2526 accessory target genes were also applied for accessory genome allelic analysis^6^. Neighbor-joining trees based on core- and accessory genome allelic profile were constructed and the corresponding pairwise distances were calculated.

### Hypermucoviscosity Phenotype Identification

Hypermucoviscosity phenotype was determined by string test. As previously described, a string test result was determined to be positive when a viscous filament greater than 5 mm in length was generated by stretching a bacterial colony with a bacteriological inoculation loop on a blood agar plate (Hadano, 2013; Shon et al., 2013).

### Serum Resistance Assay

Three independent cultures for each strain were grown overnight and diluted to 1:1000 in MH broth with or without 20% normal human serum. Three replicates of each culture were aliquoted into a flat-bottom 100-well plate. The plate was incubated at 37°C with agitation. The OD_600_ of each culture was recorded every 5 min for 10 h by using the Bioscreen C Automated Microbiology Growth Curve Analysis System (Oy Growth Curves Ab Ltd., Turku, Finland). NUTH-K2044 was employed as hvKP reference strain, while American Type Culture Collection (ATCC) 700603 as the non-hvKP reference.

### Statistical Analysis

Comparison between continuous variables (allelic gene difference) was performed by the Mann-Whitney test, as they were not normally distributed. In serum resistance assay, records for OD_600_ at each time point were summarized as mean plus 95% confidence interval.

## Results

### Characteristics of the Strain

**FIGURE 1:**
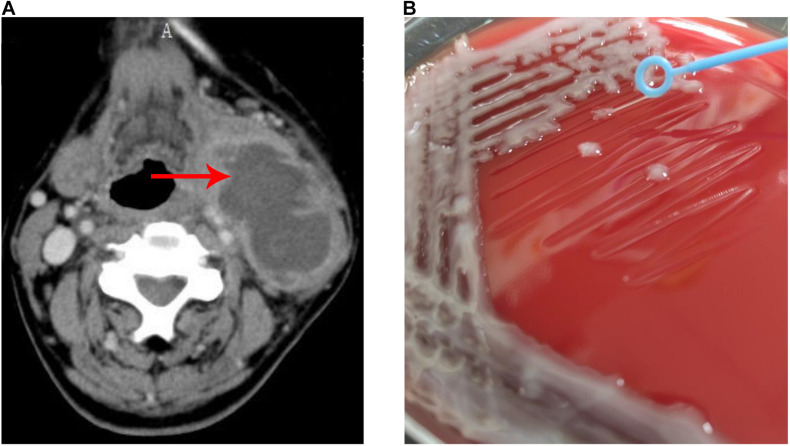
Neck abscess caused by *K. pneumoniae* strain Kp_whw. **(A)** Computer tomography image showed an abscess (66 × 42 mm) on the left side of neck. **(B)** String test for the strain Kp_whw.

A 74-year-old man, with predisposed type II diabetes mellitus and schistosomiasis cirrhosis, presented with fever and painful swelling of left neck for 1 week before admission. Computed tomographic scan revealed an abscess (66 mm × 42 mm) on the left side of neck (Figure 1A). During this hospitalization, both cultures from the neck abscess and blood yielded *K. pneumoniae*. The two strains were later determined as the same via whole genome sequencing. The patient was eventually discharged home in good condition.

**FIGURE 2:**
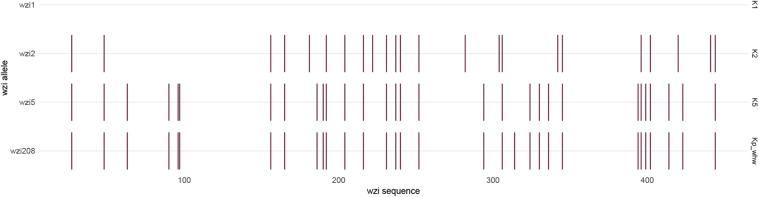
Single nucleotide variant differences of *wzi* sequences, in relation to the reference sequence *wzi1*, which is associated with K1 serotype. Each variant is indicated by a small vertical line. The *wzi* sequences are 447 bp in length. The sequences with associated locus numbers were obtain from Institut Pasteur. Kp_whw is associated with *wzi208*.

The strain Kp_whw was resistant to ampicillin but susceptible to the majority of the other antimicrobial agents, including cephalosporins, quinolones and carbapenems (Table 1). KP_whw looked shiny and cream-colored on blood agar and showed hypermucoviscosity phenotype with positive string test (Figure 1B). The strain belonged to ST 1049 and KL 5. We compared the *wzi* locus of this strain (*wzi208*) with other known loci associated with hypervirulent serotype (*wzi1*, *wzi2*, and *wzi5*) (Figure 2). The length of *wzi208* sequence is 447 bp and there were 31 SNPs difference in comparison with *wzi1* and 24 SNPs with *wzi2*. However, *wzi208* is closest to *wzi5* (associated with K5 type), with only one SNP (C314A) (Figure 2).

**TABLE 1:** Susceptibility of Kp_whw to antimicrobial agents.

Antimicrobial agent	MIC (μg/ml)	Interpretation
Ampicillin	16	R
Piperacillin	≤4	S
Amoxicillin/Clavulanic acid	≤4/2	S
Ampicillin/Sulbactam	≤4/2	S
Piperacillin/Tazobactam	≤4/4	S
Ceftazidime	≤1	S
Cefotaxime	≤1	S
Cefepime	≤2	S
Aztreonam	≤2	S
Imipenem	≤1	S
Amikacin	≤8	S
Gentamycin	≤2	S
Ciprofloxacin	≤0.5	S
Levofloxacin	≤1	S
Sulfamethoxazole-trimethoprim	≤0.5/9.5	S
Chloramphenicol	≤4	S
Tetracycline	≤2	S
Cefperazone/Sulbactam	≤8	S
Cefuroxime	≤8	S
Meropenem	≤1	S

*MIC, minimum inhibitory concentration; R, resistant; S, susceptible.*

### hvKP of K1/K2 Serotype

**FIGURE 3:**
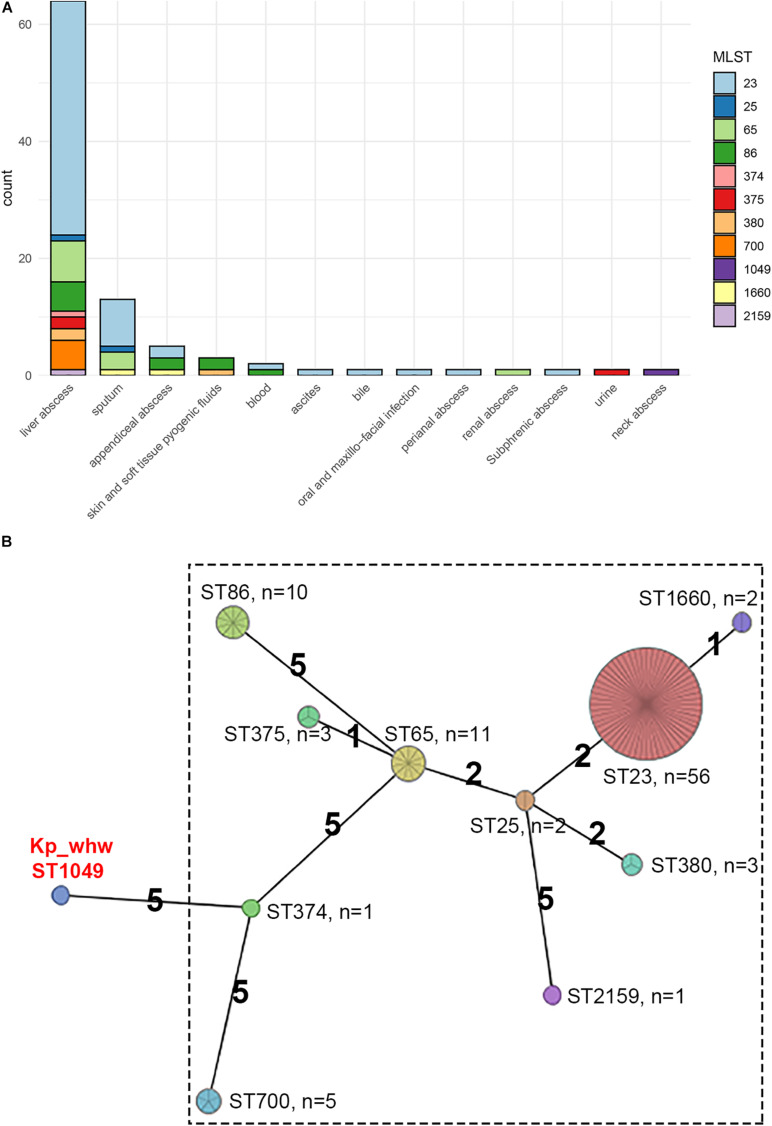
Additional 94 K1/K2 serotype hvKP strains compared with Kp_whw. **(A)** Distribution of specimen sources of the 95 strains (including Kp_whw) according to sequence type. **(B)** Minimum spanning tree (MST) of the 95 strains based on multilocus sequence typing (MLST).

The genetic similarity of strain Kp_whw with other K1/K2 serotype hvKP strains was analyzed. A total of 94 hvKP strains of K1/K2 serotype (K1, *n* = 64; K2, *n* = 30) were included. Of the 95 *K. pneumoniae* strains including Kp_whw, more than half of the strains were isolated from liver abscess (65/95, 67.4%) and 13 (13.7%) were from sputum (Figure 3A). Of note, the two isolated from bloodstream were strain NTUH-K2044 (ST23, K1 serotype, causing liver abscess and metastatic meningitis) (Wu et al., 2009) and strain hvKP1 (causing liver abscess with metastatic spread to the spleen, ST86, K2 serotype) (Russo and Gill, 2013). Similarly, Kp_whw caused invasive infections i.e., neck abscess and bloodstream infection. Minimum spanning tree (MST) based on MLST showed the relationship between strain Kp_whw and other K1/K2 serotype hvKP (Figure 3B). ST23, ST65, and ST86 were the top three clones of hvKP. Compared with ST374 (K2), strain Kp_whw had five variant loci.

### Virulence Profile

**FIGURE 4:**
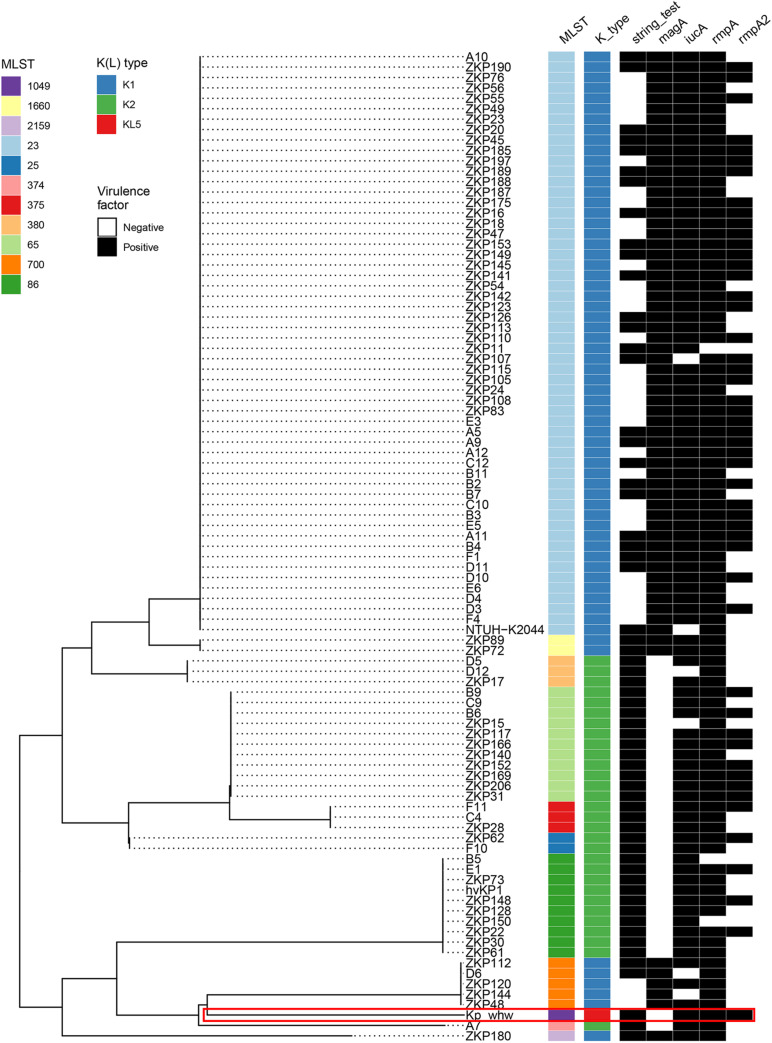
Virulence profile of the 95 hvKP strains including Kp_whw. A MLST-based phylogenetic tree was constructed for all strains. ST and K(L) type were colored according to inset legend. The presence of the virulence factors including positive string test, *magA* gene, *iucA* gene, *rmpA*, and *rmpA2* genes were indicated as black rectangles. The position of Kp_whw was indicated with a red box.

Most K1 serotype strains were ST23 (56/64, 87.5%), while those K2 serotype showed more diversity, with 11 ST65 (36.7%), 10 ST86 (33.3%), 3 ST375 (10.0%), 3 ST380 (10.0%), 2 ST25 (6.7%), and 1 ST374 (3.3%) (Figure 4). Mucoviscosity-associated gene A (*magA*), which contributes largely to invasive infection but is specific to K1 serotype (Fang et al., 2004; Chuang et al., 2006), was not detected in Kp_whw. However, both regulator of mucoid phenotype A (*rmpA*) and *rmpA2* genes were present in Kp_whw. All the K2 type strains and Kp_whw showed positive string test, while less than a half of K1 type strain (46.9%, 30/64) exhibited hypermucoviscosity phenotype.

### Mobile Genetic Elements Carrying Virulence Determinants

**FIGURE 5:**
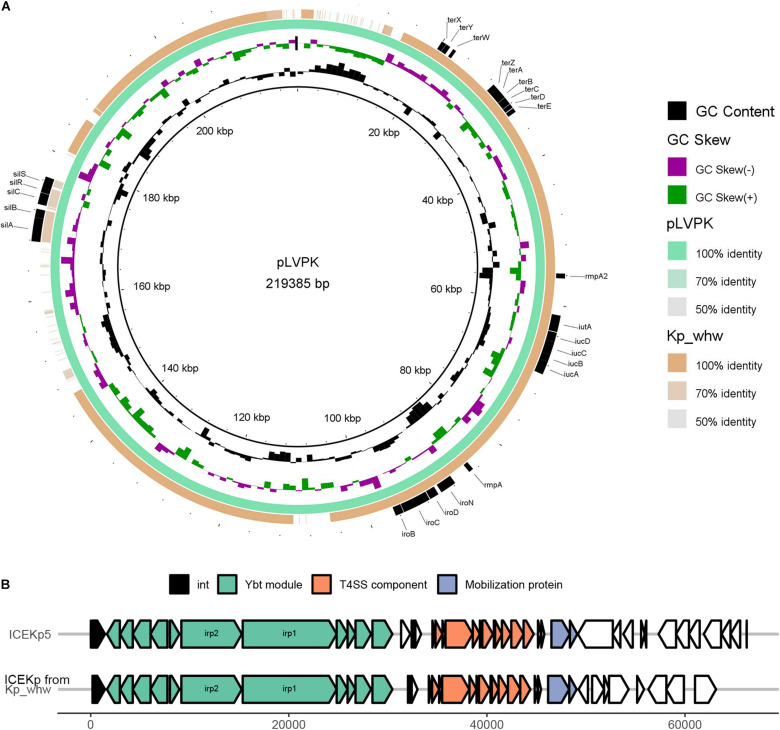
Mobile genetic elements (MGEs) carried by Kp_whw. **(A)** Alignment of the Kp_whw contigs against the virulence plasmid pLVPK (accession no. AY378100 in GenBank). Draft genome sequences of Kp_whw was aligned to pLVPK by using BLAST Ring Image Generator (BRIG). **(B)** Alignment of the putative ICE identified by ICEfinder with ICEKp5 (accession no. KY454630 in GenBank). The black arrows indicated P4-like integrase genes. Ybt module included yersiniabctin and its receptor loci. Mobilization protein was encoded by *mobB* and *mobC*.

Virulence plasmid pLVPK was well characterized previously (Chen et al., 2004). Alignment of strain Kp_whw contigs to pLVPK showed that Kp_whw carried a plasmid that aligned well to most parts of the pLVPK plasmid, including the region in which *rmpA*, *rmpA2*, and *iroBCDN* (encoding salmochelin system) and *iucABCDiutA* (encoding aerobactin system) genes were located, as well as silver and tellurite resistance gene clusters (*silABCRS* and *terABCDEXYZW*) (Figure 5A). In addition, a chromosomal ICEKp5-like mobile genetic element was detected in the strain. A *ybt* locus encoding the biosynthesis of the siderophore yersiniabactin and its receptor was mobilized by this element (Figure 5B). These findings indicated that the strain Kp_whw harbored rich virulence factors and had the genetic potential to exacerbate the dissemination of hypervirulence.

### Comparative Analysis and Phylogenetic Trees

**FIGURE 6:**
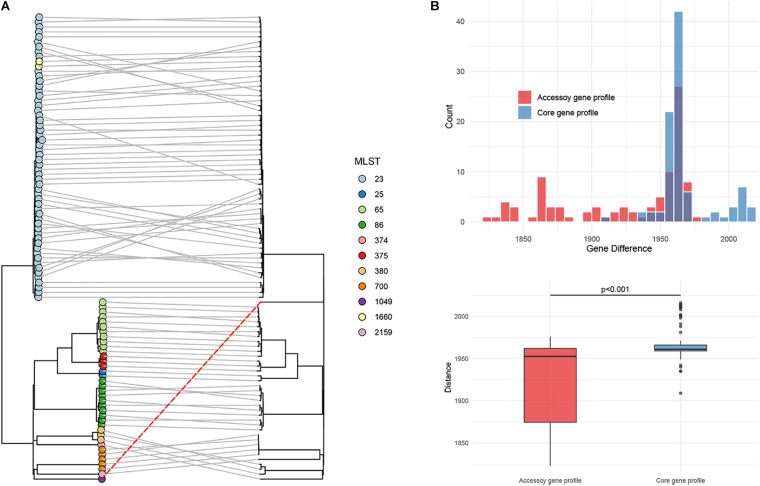
Phylogenetic analysis of the 95 hvKP strains. **(A)** The phylogeny based on accessory gene allelic profile (left) was compared with the phylogeny based on the core genomic allelic profile. Kp_whw was indiacted with a dashed red line. Sequence types were colored according to inset legend. **(B)** Allelic gene differences on accessory genomic level between Kp_whw and K1/K2 strains were compared with those of core genomic level. Allelic gene differences were calculated by Ridom SeqSphere software.

The phylogenetic tree based on accessory gene allelic profile reflected almost completely the phylogeny of the core gene alleles (Figure 6A). One notable exception to this trend was identified in Kp_whw (ST1049), which was closed to strain ZKP180 (ST2159, K1 serotype) based on accessory gene profile but unexpectedly formed an individual branch according to core gene alleles (Figure 6A). This was in accordance with the finding that allelic differences between the strain Kp_whw and K1/K2 serotype hvKP strains based on core gene profile were significantly larger than those from accessory gene profile (Figure 6B).

### Virulence Assessment

**FIGURE 7:**
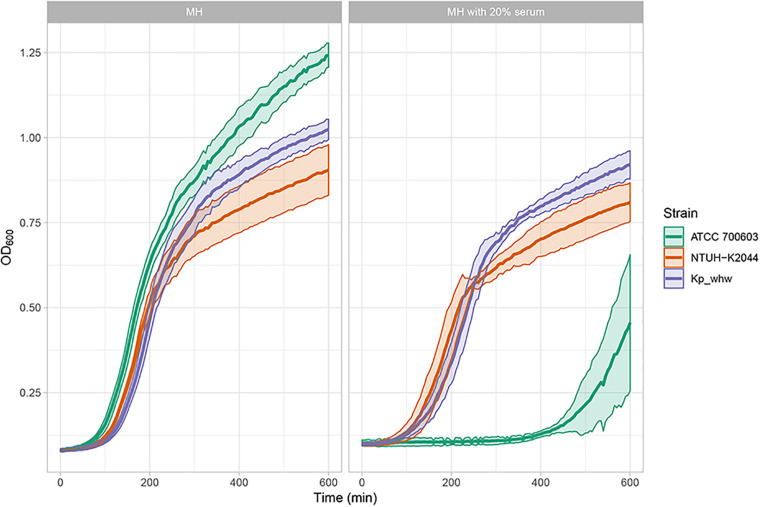
Virulence level assessment *in vitro*. As microorganisms grow, they increase the turbidity of their growth medium. By measuring the turbidity of the medium over time, an OD_600_ curve can be generated. The curve reflects the growth (increased concentration) of the organism.

To determine the virulence level of Kp_whw, serum resistant assay was performed. In MH broth without any supplement, ATCC 700603 outgrew NTUH-K2044 and Kp_whw (Figure 7). While in MH media with 20% normal human serum, however, Kp_whw and NTUH-K2044 were seldom influenced, yet ATCC700603 was dramatically inhibited to grow (Figure 7). Kp_whw and NTUH-K2044 had similar resistance to human serum, indicating that Kp_whw was as virulent as NTUH-K2044 *in vitro*.

## Discussion

Hypervirulent *K. pneumoniae* raised great concern recently. Here, we described a hvKP strain of ST1049, which was rarely reported, causing neck abscess, and invasive infection. hvKP is usually characterized as hypermucoviscosity, positive for *rmpA*, aerobactin, or virulence plasmid, and these features are frequently used to define hvKP. However, no single feature could accurately distinguish hvKP from non-hvKP (Lan et al., 2020). Clinical definition of hvKP based on the invasive liver abscess syndrome and microbiological definition of K1/K2 serotype have been accepted in recent years (Siu et al., 2012).

Neck infection is usually associated with dental procedure or oral infection (Walia et al., 2014). Although neck abscess caused by *K. pneumoniae* have been occasionally reported (Nadasy et al., 2007; Frazee et al., 2009), the genetic background and virulence level for the causative strains were seldom determined. A large retrospective analysis about deep neck abscess in China showed that *K. pneumoniae* was the most predominant gram-negative pathogen (Yang et al., 2008). Though more than a half neck abscess tended to be polymicrobial (Yang et al., 2008), most invasive soft tissue infection caused by *K. pneumoniae* were found to be monomicrobial (Park et al., 2019; Rahim et al., 2019), partly due to the survival advantages of hvKP, such as hypermucoviscosity and high siderophore production (Rahim et al., 2019). Owing to the invasiveness, Kp_whw cuased neck abscess and bloodstream infection, which was also reported with classical hvKP NTUH-K2044 (Wu et al., 2009) and hvKP1 (Russo and Gill, 2013). hvKP as a monomicrobial pathogen in invasive soft tissue infection is recently increasing and causes high morbidity and mortality (Rahim et al., 2019). Diabetes mellitus was frequently associated with *K. pneumoniae* infection (Cheng et al., 2015) and was considered as a critical risk factor (Sharma et al., 2018). The other two cases of neck abscess caused by hvKP in the United States were also associated with diabetes (Nadasy et al., 2007; Frazee et al., 2009). This might be explained by the evidence that diabetic patients have defects on the neutrophil chemotactic and phagocytic activities (Alba-Loureiro et al., 2007). Of note, both the patients were Asia descent, and one had both neck abscess and blood positive culture for *K. pneumoniae* and the other developed metastatic complications.

*Klebsiella pneumoniae* of ST1049 was seldom reported globally. An epidemiological study on liver abscess caused by *K. pneumoniae* revealed that five cases were attributed to ST1049 *K. pneumoniae* (5/163, 3.1%) (Zhang et al., 2019). However, microbiological features, virulence level, and genomic information of these strains were not analyzed. In the present study, we analyzed the K-locus for Kp_whw and determined that it belonged to KL5. The *wzi208* (a region of K-locus) for Kp_whw is closed to *wzi5*, which is associated with K5 type. In Taiwan area of China, a ST1049 strain causing meningitis also belonged to K5 serotype (Ku et al., 2017). Actually, *K. pneumoniae* of K5 serotype is also a hypervirulent clone (Holt et al., 2015) and has frequently resulted in liver abscess (Lee et al., 2016), meningitis (Ku et al., 2017), and other invasive infections (Turton et al., 2010; Guo et al., 2017). From this perspective, Kp_whw possesses hypervirulent potential. On the other hand, Kp_whw was distant from K1/K2 serotype hvKP strains according to MLST and cgMLST. *K. pneumoniae* virulence factors are encoded by genes in both the core and accessory genomes (Martin and Bachman, 2018). LPS, siderophore enterobactin and polysaccharide capsule synthesis are relative conserved and are primarily encoded by core genes while siderophore salmochelin, yersiniabactin and aerobactin are frequently ICE- or plasmid-encoded (Martin and Bachman, 2018). Compared with K1/K2 type hvKP, Kp_whw presented similar accessory genomic profile, with relative diverse allelic variation of conversed genes. These indicated that the relative higher level of core genome allelic diversity might determine the differences of genetic background between Kp_whw and hvKP strains of K1/K2 serotype.

Plasmids and ICEs are two key components contributing to HGT (Wozniak and Waldor, 2010). ICEKp is one of self-transmissible mobile genetic elements (MGEs) which encode the machinery for conjugation (type IV secretion system, T4SS) and intricate regulatory systems to control their excision from the chromosome in the host (Wozniak and Waldor, 2010). ICEKp mainly mobilizes *ybt* locus, which was first reported on the Yersinia high pathogenicity island (HPI), encoding yersiniabactin and its receptor. Yersiniabactin promoted the progression of bubonic and pneumonic plague, by scavenging iron directly from transferrin and lactoferrin (Fetherston et al., 2010). *In vivo*, yersiniabactin was demonstrated more important for *K. pneumoniae* growth than enterobactin under iron-limited conditions (Lawlor et al., 2007) and could facilitate microbes to colonize in the lungs and to cause disseminated infection (Lawlor et al., 2007; Bachman et al., 2011). Kp_whw carried an ICEKp5-like element, which containing a ybt14 locus, potentiating the transfer of yersiniabactin. Actually, additional siderophore like salmochelin (encoded by *iro* locus) can also be regulated by ICEKp (such as ICEKp1) (Lin et al., 2008). However, salmochelin, as well as aerobactin (encoded by *iuc* locus), are more frequently encoded by virulence plasmid, such as pLVPK (Chen et al., 2004). Though the affinity of aerobactin for iron is much lower than enterobactin (Brock et al., 1991), very low aerobactin concentrations are sufficient to stimulate bacterial growth (Garenaux et al., 2011), and it is essential for hvKP infection (Russo et al., 2015). For Kp_whw strain, *iuc* locus, *iro* locus and *rmpA*/*rmpA2* are simultaneously located in a single plasmid, which makes one concern about the acquisition of this type of plasmid by multi-drug resistant strains.

For a long time, virulence plasmid was deemed congenital. Carbapenem-resistant (CR) hvKP strains are increasingly emerging recently, with known mechanism of the acquisition of antimicrobial resistance genes by hvKP, but not the acquisition of virulence plasmid by CRKP, due to the non-conjugative nature of virulence plasmid (Yang et al., 2019). However, CRKP strains gaining a virulence plasmid have recently caused a lethal outbreak in China (Gu et al., 2018). It is particularly worrying that a newly reported virulence plasmid p15WZ-82_Vir could be conjugated to CRKP strains, leading to the expression of carbapenem resistance- and hypervirulence-associated phenotypes simultaneously (Yang et al., 2019). Thus, it is now our urgency to pay attention to the convergence of hypervirulence and multi-drug resistance.

In conclusion, we characterized the genetic background and virulence level of a ST1049 *K. pneumoniae* strain Kp_whw isolated from neck abscess. Kp_whw is susceptible to commonly used antimicrobial agents except for being resistant to ampicillin. Kp_whw carried a pLVPK-like virulence plasmid and harbored a ICEKp5-like mobile genetic element. All results make clear that Kp_whw is a hvKP strain and has the potential to disseminate the phenotype, which poses a substantial public health threat. Although this is the first work characterizing the genetic virulence determinants of a ST1049 hvKP strain, only one strain was analyzed. In the future, isolates with diverse clinical characteristics should be collected for deep analysis.

## Data Availability Statement

The datasets presented in this study can be found in online repositories. The names of the repository/repositories and accession number(s) can be found below: https://www.ebi.ac.uk/ena, PRJEB38367 and https://www.ebi.ac.uk/ena, PRJEB34922.

## Author Contributions

PL and DZ conceived the idea and designed the experiments. JG isolated the strain. PL and RY performed the experiments. QS analyzed the data. YJ and JZ helped with materials and reagents. PL wrote the manuscript. YY reviewed the manuscript. All authors have read and agreed to the published version of the manuscript.

## Conflict of Interest

The authors declare that the research was conducted in the absence of any commercial or financial relationships that could be construed as a potential conflict of interest.

## Footnotes

1 https://bigsdb.pasteur.fr/klebsiella/klebsiella.html

2 https://bigsdb.pasteur.fr/cgi-bin/bigsdb/bigsdb.pl?db=pubmlst_klebsiella_seqdef

3 http://db-mml.sjtu.edu.cn/ICEberg/

4 https://github.com/katholt/Kleborate

5 https://www.cgmlst.org/ncs/schema/schema/2187931/

6 https://www.cgmlst.org/ncs/schema/2192830/
